# Vocal biomarkers correlate with FEV1 variations during methacholine challenge

**DOI:** 10.1002/clt2.70055

**Published:** 2025-03-28

**Authors:** Giovanni Paoletti, Giovanni Costanzo, Morena Merigo, Francesca Puggioni, Sebastian Ferri, Maria Rita Messina, Fulvio Cordella, Giuseppe Ranieri, Arianna Arienzo, Victor Savevski, Giorgio Walter Canonica, Ayana de Brito Martins, Enrico Heffler

**Affiliations:** ^1^ Department of Biomedical Sciences Humanitas University Pieve Emanuele Italy; ^2^ Personalized Medicine, Asthma and Allergy IRCCS Humanitas Research Hospital Rozzano Italy; ^3^ VoiceMed s.à.r.l. Strassen Luxembourg; ^4^ Artificial Intelligence Center IRCCS Humanitas Research Hospital Rozzano Italy

**Keywords:** artificial intelligence, asthma, digital health, mobile health technology, vocal biomarkers

## Abstract

**Background:**

Mobile health applications are increasingly valued for their role in asthma management and the opportunity for large dataset collection. Our study aimed to investigate the feasibility of applying signal‐processing and machine‐learning technologies to detect alterations in the lower airway caliber and develop a machine‐learning algorithm to identify changes in vocal biomarkers and detect bronchoconstriction in patients with airway hyperreactivity.

**Methods:**

This is an explorative observational prospective longitudinal study focused on vocal biomarkers and their association with bronchial constriction and respiratory function. Non‐smoker adults with clinical suspicion of asthma were consecutively enrolled from May 2023 to September 2023. At each step of a Methacholine Challenge Test (MCT) performed on these patients, the respiratory sounds were recorded via a smartphone through an app specifically developed. Several biomarkers were extracted and their relationship with the change in Forced Expiratory Volume in the first second (FEV1) was measured.

**Results:**

Forty‐two subjects were enrolled. The highest correlation with FEV1 came from exhalation vocal events. No single feature exhibited robust behavior across different subjects, while each subject showed “personal” highly correlated features. All values were strongly statistically significant irrespectively of the result of MCT.

**Conclusion:**

The app’s algorithm is sensitive in correlating specific vocal biomarkers to FEV1 variations during MCT. This feature may assist physicians in diagnosing asthma and its exacerbation and in assessing therapy response and adherence. The socio‐economic implications might be significant, and the simplicity of use makes it an ideal tool for research.

## 1 INTRODUCTION

Asthma is a chronic respiratory disease characterized by variable expiratory airflow limitation causing a wide range of symptoms such as dyspnea, wheezing, shortness of breath, chest tightness, and/or cough. Those manifestations may vary over time in severity and are often triggered by specific factors such as allergen expositions, exercise, changes in weather, or viral respiratory infections. Symptoms and airflow limitation may sometimes be absent for weeks or months and resolve spontaneously or in response to medication. Nevertheless, patients can experience sudden, sometimes life‐threatening exacerbations.^1^, ^2^

Asthma is one of the most common chronic non‐communicable diseases worldwide, affecting around 300 million individuals globally, with a global prevalence that may vary from around 10% in children and adolescents and 6%–7% in adults, with higher percentages in high‐income countries.^3^, ^4^, ^5^, ^6^

Mobile health applications, or mHealth apps, are increasingly valued for their role in managing asthma. They offer several benefits for both patients and healthcare providers.

The MASK‐air app is one of the great examples of how mHealth tools can bridge the gap between patients and healthcare providers. It helps in better disease tracking and management by allowing patients to record their symptoms and medication use in real‐time. The great limitation is the use of many questionnaires, which impacts patients' compliance. This continuous stream of data can then be used to customize treatment plans. Bousquet et al.^7^ have shown that the real‐world data from the app can refine clinical guidelines and ensure treatments are more tailored to individual needs.

One significant advantage of mHealth apps is their ability to improve patient adherence to treatment regimens. Poor adherence is a common issue in asthma management, often leading to suboptimal disease control. These apps, with their interactive features, regular reminders and easy access to health information, encourage patients to follow prescribed treatments more consistently.^8^

Additionally, incorporating environmental factors such as air pollution and pollen levels into these apps provides a comprehensive approach to asthma management. Different authors have stressed the importance of including these variables, as they can significantly influence asthma symptoms. By embedding such data, apps can offer predictive insights and alert patients to potential asthma triggers, allowing for preemptive action.^9^

Using mHealth apps also supports the collection of large datasets, contributing to valuable real‐world evidence that can inform public health strategies and clinical guidelines. For example, data from the MASK‐air app was utilized to enhance the ARIA guidelines, integrating behavioral science and patient needs into asthma care protocols.^10^

In recent years the recognition that respiratory inflammation commonly affects both the upper and lower respiratory tracts, often concurrently, has increasingly arisen. Therefore the upper and lower airways must be viewed as an integrated system.^11^ Considering this new point of view, recent studies found a connection between vocal symptoms and allergy or asthma in adults and children.^12^ In a small study involving 49 young students, subjects with allergies reported significantly more vocal symptoms than subjects without allergies.^13^ In other studies, people with pollen allergy have been shown to experience more vocal symptoms than controls during and between pollen seasons, with an increase of vocal symptoms during the pollen season and a decrease during the non‐pollen season.^14^ Moreover, patients with more air‐borne allergies have a higher incidence of undiagnosed vocal dysfunction.^15^

Different features of the allergic respiratory disease may lead to vocal symptoms. In 2010 the German otolaryngologist Hackenberg and colleagues^16^ reported that half of the patients with asthma complained about permanent voice disorders: according to the Authors, this may be the consequence of the inflammation itself (mucosal changes, mucus abnormalities, the frequently associated chronic rhinosinusitis), or, as suggested in other articles,^17^, ^18^ a side effect of the background therapy (inhaled corticosteroids‐induced chronic laryngitis). Moreover, vocal symptoms may also be caused by the exposition to the allergen itself, as a study by Dworkin et al. suggested: allergen provocation of the larynx may led to viscous secretions in the larynx, vocal fold edema and erythema, coughing, throat clearing, and hoarseness in people with perennial dust mite allergy.^19^

Vocal changes have been reported in many non‐respiratory diseases as well. Indeed, the evolution of voice technology has led to the identification of vocal biomarkers for diagnosis, classification, or patient remote monitoring, or to enhance clinical practice.^20^

A biomarker is an objectively measured and evaluated characteristic (anatomical, serological, or physiological) used to indicate a normal biological process, a pathological process, or a biological response to a therapeutic intervention.^21^, ^22^

A vocal biomarker is a signature, a feature, or a combination of features in the voice that has been identified and validated as associated with a clinical outcome.^23^ Work on vocal biomarkers has mainly been performed in the field of neurodegenerative disorders, like Parkinson's disease,^24^, ^25^ Alzheimer's disease,^26^, ^27^ mild cognitive impairment,^28^ and multiple sclerosis.^29^ In recent times, likely in conjunction with the coronavirus Disease 19 (COVID‐19) pandemic, research investigating vocal biomarker association with respiratory disorders has received good momentum.^30^, ^31^, ^32^, ^33^, ^34^

Although asthma is a heterogeneous disease with multiple clinical presentations, it affects the same system and structures used for voice production. Abnormalities in vocal production processes can lead to changes in voice quality.

Phoneticians and voice clinicians are particularly interested in using acoustic analysis to understand voice quality as this method is non‐invasive, affordable, and easy to use. Furthermore, machine learning algorithms have demonstrated their ability to identify features in vocal sounds related to abnormalities in the vocal system.

Our study aimed to investigate the feasibility of applying signal‐processing and machine‐learning technologies to detect alterations in the lower airway caliber of the lower respiratory tract and develop a machine‐learning algorithm capable of identifying changes in vocal biomarkers that can be used for monitoring bronchoconstriction in patients with airway hyperreactivity.

For this purpose, the outcomes of our study were:

- To compose a dataset for mapping smartphone‐acquired voice recordings to pulmonary function as measured by spirometry.
- To identify respiratory function‐correlated acoustic biomarkers that may help in anticipating severe alterations in the bronchial caliber and therefore in the respiratory function itself.
- To evaluate the goodness of smartphone‐acquired voice recordings analysis for further development and study of a machine learning‐based algorithm with the above feature as input that may identify alterations in the caliber of the lower airways.

## 2 MATERIAL AND METHODS

This study is an explorative observational prospective longitudinal study, aiming to fill the gap in knowledge about vocal biomarkers and their association with bronchial constriction and respiratory function. Non‐smoker adults (age ≥ 18 years) without a confirmed diagnosis of asthma, but to whom a methacholine challenge test (MCT) was prescribed, were consecutively enrolled from May 2023 until September 2023. Patients with symptoms and/or signs consistent with upper and/or lower airway infections, and those with classical contraindication for MCT (i.e.: myocardial infarction or stroke in last 3 months, uncontrolled hypertension, aortic aneurysm, recent eye surgery, or intracranial pressure elevation risk)^35^ were excluded from the study and did not performed MCT. All subjects were capable of understanding the written informed consent form, agreeing to comply with protocol requirements, and providing signed and witnessed informed consent. Subjects with severe sensory impairments, such as visual and auditory impairments, were excluded. All subjects compiled a Case Report Form with several questions about past and current diagnosis, smoking habits and other metadata in order to better contextualize each collected datapoint. Subjects who answered positively to at least one of the following questions were considered atopics:

- Have you ever been diagnosed with allergic rhinitis?
- Have you ever been diagnosed with allergic asthma?
- Have you ever been diagnosed with allergic aspergillosis?
- Have you ever been diagnosed with other respiratory diseases/conditions?

### 2.1 Spirometry and methacholine challenge test (MCT)

All patients were evaluated for lung function parameters by means of a portable spirometer (CareFusion®). Patients with evidence of obstructive pattern at the spirometry were excluded from the study, as for them, MCT was contraindicated according to international recommendations.^35^

Patients with normal lung function underwent MCT.

Calibrated nebulizers to an output of 0.010 mL per inhalation (MB3; Mefar) were used to administer saline or methacholine. For safety reasons, the test started with two breaths of normal saline (NaCl 0.9%), followed by a pause of 120 s before performing the second spirometry. If the FEV_1_ dropped >20%, the test was discontinued. Otherwise, methacholine chloride was administered by inhalation through the nebulizer at an initial dose of 100 mcg. After inhalation of the aerosol, FEV_1_ was measured after 120 s with two spirometric maneuvers. The amount of methacholine was sequentially increased in doubling‐dose manner until a decrease in FEV_1_ greater than 20% was observed or up to the cumulative dose of 1600 mcg. The provocative dose of methacholine causing a 20% drop in FEV_1_ (PD20FEV1) was recorded, and if it was ≤800 mcg the MCT was considered positive.

### 2.2 Respiratory sounds recording

The respiratory sounds were recorded via smartphone through a specifically created app at each test MCT step. Participants were provided with instructions to download the study smartphone app and a unique code and password. This app was developed by VoiceMed, a startup that is developing an algorithm for detecting signs of airway narrowing in sound recordings. Sound recordings were recorded through the app containing a module developed specifically for this study. The sounds recorded through the patient app were combined with clinical data. This mapping between sound recordings and clinical and demographic data was the basis for developing the proposed algorithm. In addition to documenting the eligibility criteria, the investigators collected also the following information about the patient: smoking history, physical activity, relevant previously diagnosed conditions, and whether the asthma diagnosis was subsequently confirmed. The following information from the spirometry results was paired to the app for statistical analysis: Age (in years); Height; Weight; Sex; Date and time of measurement; Flow and volume measurement (FVC, FEV_1_, FEV,/FVC, FEF_25–75_).

The respiratory task involved three cycles of deep inhalation and exhalation with open mouths followed by a deep inhalation from the nose and the production of a vocalization characterized by an “A” as long as possible. The dataset was organized and analyzed through audio analysis software. Each audio track has been manually checked indicating the recording quality and the time position of the respiratory events of interest. In case of insufficient quality, the software algorithm automatically ignored the recording. The code has been programmed to segment and group the sound events, pre‐process them in order to enhance the acoustic resonances and extract 40 different parameters (further details in Table 1). In the case of the vowels, each parameter has been computed at different time steps and converted to mean values and standard deviation on the phonation time, while for the respiratory events, parameters have been computed once for each event and converted to mean values and standard deviation on the three repetitions. Hence, a total of 80 biomarkers (40 average values and 40 variation values) per sound type have been collected. Hereafter, the notation μ_*n*_–σ_*n*_ relatively for average value and variation value of parameter “*n*” is used.

**TABLE 1:** List of computed parameters.

Biomarker ID	Name	Description
1	Duration	The length of the event in seconds
2	Breathing rate ‐ Pitch	The occurrence frequency of the respiratory event in beats per minute—For the vowel, the oscillation frequency of the vocal folds in Hertz
3	Spectral centroid	Mean frequency of the intensity‐frequency distribution. It roughly tells the average shift of the spectral activity.
4	Spectral spread	Standard deviation of the intensity‐frequency distribution. It roughly tells the average frequency bandwidth of the spectral activity.
5	Spectral energy	Area of the intensity‐frequency distribution. It's the measured acoustic intensity.
6	Spectral flatness	Geometric mean/arithmetic mean ratio of the intensity‐frequency distribution. It measures the degree of harmonicity/airness.
7	Spectral roll‐off‐80	The limit frequency up to which 80% of the total intensity is measured. It roughly measures the concentration of the frequency content in the lower‐end.
8	Spectral slope	The average intensity decay/rise across frequency. It roughly measures the ratio between low‐frequency and high‐frequency intensity.
9	Spectral exponent	The average intensity decay/rise across frequency on the log‐scaled spectrum. It roughly measures the ratio between low‐frequency and high‐frequency intensity in logarithmic scale.
10–23	Linear prediction coefficient 1–14	One of the parameters of the spectrum's polynomial representation. It partially compresses the resonance’s location and width.
24–40	Mel Frequency cepstral coefficient 1–17	Intensity of relative component of the mel‐encoded spectrum in logarithmic scale. It partially compresses the information about vocal‐tract shape.

*Note*: This set is highly focused on resonance representation and spectral shape representation on different levels of complexity and generalization.

### 2.3 Sound processing algorithms

A sound quality evaluator analyzed each sound signal to assess its quality. Whenever a signal did not meet the quality requirements, it was discarded, on the contrary, the audio file was sent to further algorithms whose description follows.

Given a specific vocal task, such as a respiratory task, the evolution of airflow dynamics inside and outside the human system produces specific vibrations in the audible spectrum. These vibrations travel across the whole respiratory tract, suffering from many reflections/transmissions/absorptions due to the complex geometry of the cavity, the surrounding tissue, and the side muscular activity. Given these basics, the lower airways component is expected to suffer from a significant reduction in terms of intensity. In this regard, a set of algorithms was used to enhance the resonant content depenalizing the weakest harmonics to amplify them against the strongest ones. Finally, the pre‐processed audio was sent to further algorithms for the extraction of several features.

**FIGURE 1:**
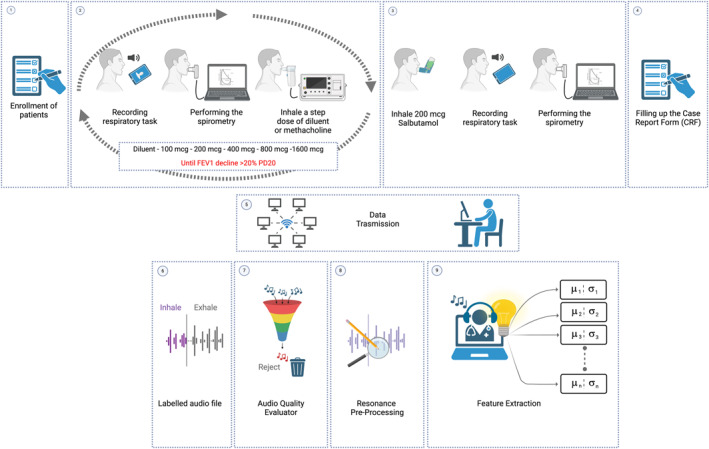
Study procedures. Patients were enrolled according to inclusion and exclusion criteria, and after having signed the informed consent (1). Respiratory tasks were recorded, spirometry was performed, and the dose of diluent or methacholine was inhaled. This was repeated until the patient had a 20% reduction in FEV1 compared to the baseline spirometry or up to the maximum methacholine dose according to the protocol (2). Then, patients inhaled salbutamol, and if the patient has had a positive methacholine, repeated at intervals of 15 min both recording of respiratory task and the spirometry (3). Patient filled in the questionnaire (4) and all collected data were anonymously transferred to the electronic case report form directly from the mobile phone app (5). The input file was contextualized with time labels so that every further step was applied to the right portion of audio (6). The audio quality evaluator computed several acoustic measurements in order to validate the audio file (7). In case of wrong performance, interrupted or noisy audio, the file was rejected. Then, the pre‐processing step was applied to each event in order to enhance its properties (8). Finally, the feature extraction block fetched all the described parameters from the pre‐processed audio file and sent them to further analysis. *Source:* This figure was created with BioRender.com.

These features were the main analysis items and were expected to vary across the test procedure in relation to narrowing phenomena and were extracted from manually labeled respiratory tasks only, avoiding silence and any noisy parts present in the audio file. A summary of the flowchart of the study is shown in Figure 1.

The vast majority of computed biomarkers are derived from the frequency spectrum of the waveform, which represents the strength of each acoustic frequency in the analyzed signal, resulting in an intensity‐frequency distribution. To obtain this spectrum, the signal was processed using the Fourier Transform, a mathematical technique that converts a time‐domain signal into its frequency‐domain representation. Given a sampling rate of 44.1 kHz, the frequency spectrum is limited to 22.05 kHz, as dictated by the Nyquist theorem, thus the implemented algorithm is blind to frequencies above such a limit. Several mathematical operations were performed on the frequency spectrum to derive more concise and effective measures, resulting in the list of biomarkers shown in Table 1.

### 2.4 Statistical analysis

The descriptive power of each biomarker has been studied through the Pearson correlation test with FEV_1_ as the target variable. Specifically, in order to avoid possible biases due to sex and age distribution that are well known to have a significant impact on voice, the intra‐subject correlation has been observed, comparing each subject's biomarker‐trace with the relative subjects's FEV_1_ trace across the test.

Pearson's correlation coefficient (*ρ*) measures the linear relationship between each biomarker and the target variable: a strong correlation (*ρ* close to 1 or −1) means a high degree of direct explainability from the first variable to the second; a low correlation (*ρ* close to 0) means that both variables are not directly influenced by each other. For these reasons, the absolute value |*ρ*| has been finally used to evaluate each biomarker, ignoring the direction of the correlation.

In case of high values of |*ρ*| between one of the vocal biomarkers and the subject's FEV_1_ trace, it would mean that every biomarker data‐point closely matches every FEV_1_ data‐point not necessarily in terms of value but in terms of variation (i.e. a FEV_1_ fall of 20% matched with a biomarker fall/rise of 20% means roughly a good |*ρ*| regardless of the actual FEV_1_/biomarker value).

### 2.5 Ethical considerations

The study was conducted according to the principles of the International Council for Harmonisation of Technical Requirements for Pharmaceuticals for Human Use (ICH) guideline for good clinical practice (E6 R2) and with approval of the IRCCS Humanitas independent ethical committee (protocol number 3472, date May 05, 2023). The study was conducted in accordance with all national, state, and local laws and regulations, good clinical practices (decreto ministeriale 15/07/1997 and subsequent amendments and additions), the Oviedo Convention (4th of April 1997), and its additional protocol (CETS No.195, 25th of January 2005) as well as the last amendment of the World Medical Association Declaration of Helsinki (October 2013). All enrolled patients signed a written informed consent for the participation in the present study.

## 3 RESULTS

Forty‐two subjects were enrolled. Two were excluded due to technical issues in acquiring the audio recording, leading to 40 analyzed subjects (23 females, 57.5%) and 256 total audio files. Twenty‐five patients (62.5%) were atopic, 3 (7.5%) smokers, 12 (30.0%) ex‐smokers, and 8 (20.0%) reported to be affected by any upper airway inflammatory disease (such as rhinitis, rhinosinusitis with or without nasal polyps, adenoiditis…). The mean BMI was 23.28 ± 4.46 kg/m^2^. In 13 patients (32.5%) MCT resulted positive for airway hyperreactivity, with a mean PD_20_FEV_1_ of 530.77 ± 264.24 mcg. Table 2 summarizes demographic, clinical and lung function features of enrolled patients.

**TABLE 2:** Demographic and lung function characteristics of the enrolled patients.

Parameter	Characteristic
Age (years ± SD)	41 ± 12
Sex (females, %)	23, 57.5
BMI (kg/m^2^ ± SD)	23.28 ± 4.46
Basal FEV_1_ (*L* ± SD)	3.40 ± 0.79
Basal FEV_1_% predicted (% ± SD)	99.30 ± 12.09
Basal FVC (*L* ± SD)	4.29 ± 0.97
Basal FVC % predicted (% ± SD)	102.20 ± 12.31
Basal FEV_1_/FVC ratio (% ± SD)	79.57 ± 8.26
Basal FEF_25–75_ (*L/s,* ± SD)	3.29 ± 1.26
Basal FEF_25–75_% predicted (% ± SD)	94.00 ± 28.93
PD_20_FEV_1_ (mcg ± SD) (of 13 positive individuals)	530.77 ± 264.24

**FIGURE 2:**
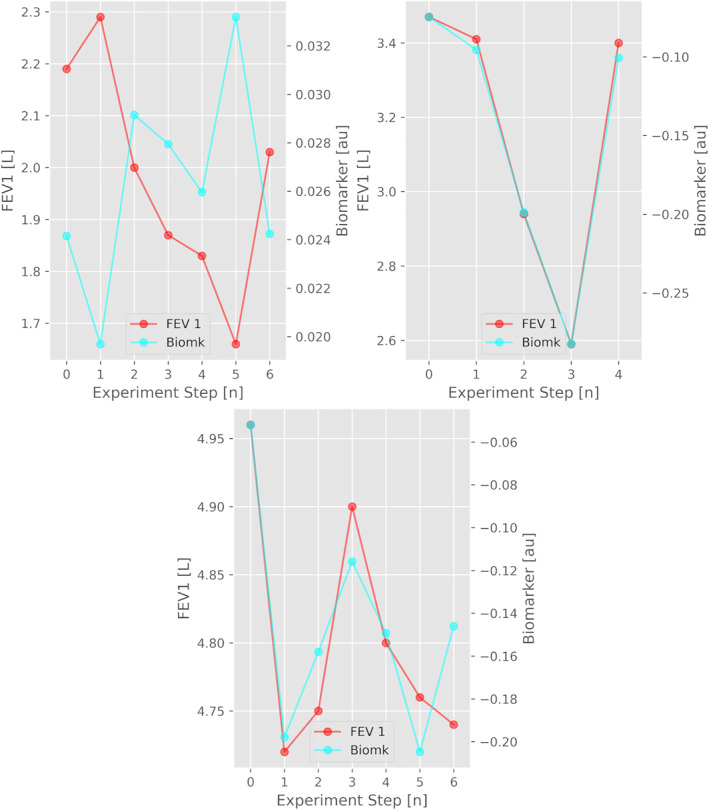
Overlapped traces of FEV1 and biomarkers for subject 30 (top left), subject 39 (top right) and subject 5 (bottom). It's worth noting that: (upper‐left panel) subject 30’s FEV1 trace is inversely correlated with relative biomarker; (upper‐right panel) subject 30 and 39’ traces are consistently followed even at the last step of the test, which for both subjects was the salbutamol dose for pulmonary function recovery, where each biomarker showed a variation towards the baseline value of the first step; (lower panel) subject 5 resulted negative to the test, having moderate, above‐threshold, variations of FEV1 across the procedure but still with a biomarker that followed those variations in a consistent way.

The highest correlation with FEV_1_ came from exhalation vocal events, for this reason only results from this type of event were treated. None of the features listed in Table 1 exhibited robust behavior across different subjects as the best value was |*ρ*| = 0.53 ± 0.26 for μ_40_, while each subject showed his/her own “personal” highly correlated features: the subject with the lowest maximal correlation with FEV_1_ was subject n.30 with feature μ_15_ (|*ρ*| = 0.78, *p* < 0.05), while the greatest correlation was reached by subject n.39 with μ_39_ (|*ρ*| = 0.99, *p* < 0.01). Figure 2 shows their relative behavior across the test. A more comprehensive overview is reported in Table 3 where best correlation values of all subjects along with relative parameters are presented. As shown all values were strongly statistically significant irrespectively of the result of MCT. It's worth noting that each “best‐biomarker” is an average μ_*n*_ and no variation value σ_*n*_ is in Table 3, suggesting that the respiratory component is stronger in the actual value of the biomarker rather than its variability across several repetitions. Inhalation and vocalization showed a non‐relevant |*ρ*| range in every parameter (maximum correlation was below 0.5).

**TABLE 3:** List of subjects and their best biomarker with relative correlation values and statistical significance.

Subject	Parameter	*|ρ|*	*p*‐value	MCT Result
1	μ_27_	0.95	<0.01	Negative
2	μ_24_	0.99	<0.01	Negative
3	μ_32_	0.95	<0.01	Negative
4	μ_30_	0.96	<0.01	Negative
5	μ_29_	0.89	<0.01	Negative
6	μ_33_	0.98	<0.01	Negative
7	μ_5_	0.99	<0.01	Negative
8	μ_11_	0.88	<0.01	Negative
9	μ_32_	0.89	<0.01	Negative
10	μ_4_	0.94	<0.01	Negative
11	μ_13_	0.99	<0.01	Positive
12	μ_23_	0.93	<0.01	Positive
13	μ_25_	0.99	<0.01	Positive
14	μ_40_	0.99	<0.01	Negative
15	μ_19_	0.96	<0.01	Negative
16	μ_3_	0.99	<0.01	Negative
17	μ_15_	0.91	<0.01	Negative
18	μ_6_	0.90	<0.01	Negative
19	μ_7_	0.98	<0.01	Positive
20	μ_3_	0.99	<0.01	Negative
21	μ_18_	0.96	<0.01	Positive
22	μ_39_	0.99	<0.01	Negative
23	μ_8_	0.99	<0.01	Positive
24	μ_23_	0.99	<0.01	Positive
25	μ_7_	0.94	<0.01	Negative
26	μ_21_	0.97	<0.01	Positive
27	μ_26_	0.92	<0.01	Negative
28	μ_38_	0.96	<0.01	Positive
29	μ_11_	0.99	<0.01	Negative
30	μ_15_	0.78	<0.05	Positive
31	μ_37_	0.93	<0.01	Negative
32	μ_3_	0.98	<0.01	Positive
33	μ_23_	0.98	<0.01	Negative
34	μ_9_	0.94	<0.01	Negative
35	μ_4_	0.89	<0.01	Negative
36	μ_22_	0.97	<0.01	Positive
37	μ_15_	0.89	<0.01	Negative
38	μ_36_	0.97	<0.01	Negative
39	μ_39_	0.99	<0.01	Positive
40	μ_16_	0.95	<0.01	Negative

*Note*: On a personal basis, each listed biomarker looks promising for pulmonary function tracking with consistent correlation values paired with robust statistical significance. Knowing in advance the best parameter for every subject, would have meant an accurate detection of significant FEV_1_ falls and recovery.

## 4 DISCUSSION

The development of automated acoustic sound analysis, capable of capturing breath, voice, cough, wheeze, and inhaler use, presents a promising opportunity for enhancing the diagnosis and monitoring of asthma. The systematic review carried out by K. Wieczorek and colleagues has underscored the significant potential of acoustic biomarkers, particularly cough and wheeze, in assisting the diagnosis and monitoring of asthma. The findings indicate the prospect for the clinical integration of acoustic biomarkers, highlighting the necessity for further validation in larger and more diverse clinical populations.^36^

Rogers and colleagues conducted a review that synthesizes the current knowledge on the application of artificial intelligence (AI) in analyzing pediatric voice as a biomarker for health. The review revealed a global representation of pediatric voice studies, focusing on developmental, respiratory, speech, and language conditions. The most frequently studied conditions were autism spectrum disorder, intellectual disabilities, asphyxia, and asthma. The analysis of pediatric voice using AI shows promise as a non‐invasive, cost‐effective biomarker for a wide range of pediatric conditions.^37^

**FIGURE 3:**
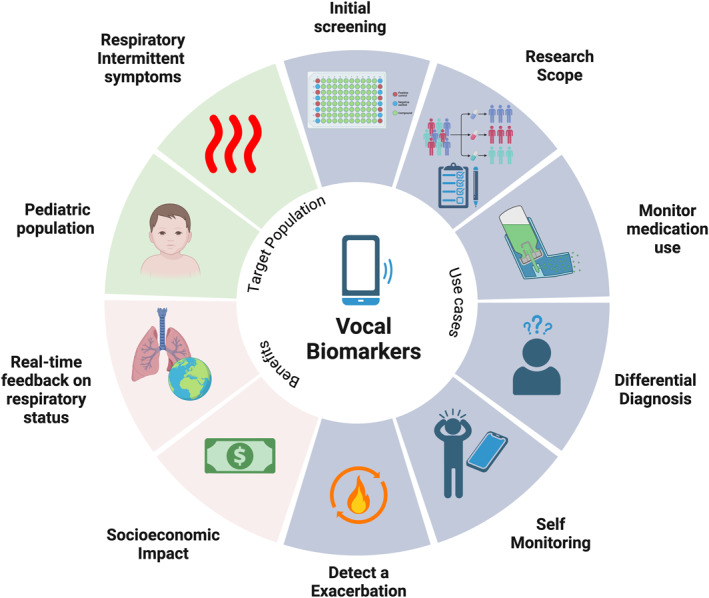
This figure summarizes the potential use of vocal biomarkers developed to detect bronchial obstruction when integrated into apps and connected systems designed for asthma care and management. These markers can enhance respiratory screening campaigns, provide real‐time feedback to patients about their respiratory status, and monitor medication use. Furthermore, they can support the differential diagnosis of dyspnea, enables effective self‐monitoring, and helps identify exacerbations early. By reducing both direct and indirect asthma‐related costs and offering valuable research insights, their use represents a significant advancement in asthma care and management. They could be especially beneficial also for children, patients with intermittent respiratory symptoms, and patients with impaired perception of airflow obstruction. *Source:* This figure was created with BioRender.com.

Based on the evidence in literature, in this study we recorded various vocal biomarkers collected in 40 patients performed during each step of the MCT (13 tested positive for airway hyperreactivity). Our goal was to determine if there was a correlation between specific vocal biomarkers and the reduction of FEV_1_. Our results indicate that our app’s algorithm is highly sensitive in correlating specific vocal biomarkers to FEV_1_ variations during exposure to methacholine, irrespectively of the final result of the MCT. Variable airway obstruction is one of the most characterizing clinical feature of asthma and acute bronchospasm is responsible of the onset of typical symptoms that might be also potentially life‐threatening.^38^ Therefore, a non invasive, easy and rapid to use tool capable of rapidly identifying bronchospasm would be of great assistance in every phase of the management of a complex pathology such as asthma, from the diagnosis to the assessment of exacerbations, up to the evaluation of therapeutic response. Such a tool can be incorporated into mobile phone apps designed to assist clinicians in diagnosing and monitoring airway obstructive diseases, particularly asthma, and patients, in increasing awareness of their disease, its burden, and the impact of the therapy (see Figure 3).

The development of a digital tool capable of detecting bronchial obstruction can have significant implications for clinical practice. This tool can help in the early and non‐invasive detection of bronchial obstruction, enabling timely intervention. Additionally, an app’s portability and ease of use make it a practical solution for use in various settings, including at home and in primary care settings, reducing the need for hospital visits at the occasions when it is appropriate and thus dropping associated healthcare costs.

An app’s ability to detect bronchial obstruction positions it as a potential tool in the diagnostic process. General practitioners often face the challenge of distinguishing between different respiratory conditions and determining the need for specialist referral during the initial screening. An accurate and easy‐to‐use diagnostic tool can aid in this decision‐making process in diseases, like asthma and chronic obstructive pulmonary diseases (COPD) that are often misdiagnosed bot at primary and secondary health‐care levels.^39^

By providing real‐time data on bronchial obstruction, it can help identify patients who require further testing and specialist intervention, improving the efficiency of the diagnostic process. This can ensure that patients receive appropriate care promptly and reduce the burden on healthcare systems by minimizing unnecessary specialist referrals.

One of the app’s most significant advantages is its easy and rapid application. Traditional spirometry, while effective, can detect airway obstruction only when the feature is only when bronchospasm is ongoing.^40^ Despite being a key feature of asthma, bronchial obstruction is not always present, especially if the disease is in its earliest stages and the patient’s characteristics allow for appropriate functional performance. Therefore, spirometry may not always capture transient bronchospasms that patients, particularly younger ones, experience only in response to specific triggers.^41^ The ability of the app to rapidly detect bronchial obstruction when it strikes addresses this limitation, offering a dynamic assessment of the patient’s condition.

This functionality is crucial in identifying bronchospasm episodes that may occur outside clinical settings, thus providing a more comprehensive understanding of the patient’s respiratory health. It also allows for the immediate direction of patients directly toward more specific tests, such as bronchodilation or methacholine challenge tests, which can confirm the presence of bronchial hyperreactivity.

The differential diagnosis of dyspnea can be challenging, particularly in distinguishing between bronchospasm‐related dyspnea and other causes such as cardiac conditions, anxiety, or upper airway obstructions. An app with such a feature can play a pivotal role in the initial evaluation by providing objective data on bronchial obstruction.^42^

The app could help in the differential diagnosis of bronchospasm in general: it may assist in distinguishing this potentially rapidly dangerous condition from other diseases sometimes characterized by a similar clinical manifestation but unable to put the patient in immediate severe harm, such as hyperventilation in panic disorder. On the other hand, it may help in distinguishing an airway obstruction from other severe immediate‐referral‐requiring diseases that manifest themselves with dyspnea as well but are caused by completely different pathogenesis and, therefore, require completely different diagnostic and therapeutic management, such as pulmonary embolism.

By integrating these vocal biomarkers into the diagnostic pathway, clinicians can quickly determine whether bronchospasm contributes to the patient’s symptoms. This can streamline the diagnostic process, reduce the time to diagnosis, and allow for more targeted and effective treatment plans. Moreover, it can help alleviate patient anxiety by providing clear and immediate insights into their respiratory status.

Asthma management is a complex process that requires continuous assessments to ensure optimal control of the condition. A new app capable of detecting bronchial obstruction has significant potential to enhance asthma monitoring.^43^

Another potential benefit is its capability for patient self‐monitoring. Self‐monitoring tools empower patients by providing them with real‐time feedback on their respiratory status. This increased awareness can significantly enhance their understanding of the disease and its triggers, leading to better management of their condition.

Studies have shown that patients who actively engage in self‐monitoring are more likely to adhere to their therapeutic regimens. Monitoring bronchial obstruction at home allows patients to recognize early signs of exacerbation, prompting timely intervention.^44^ This proactive approach can prevent severe asthma attacks and reduce the need for emergency medical care and the resulting cost^45^

Furthermore, GINA recommendations suggest ICS‐LABA as needed in each phase, or step, of asthma therapeutical management.^46^ A tool capable of helping the patient recognize a real bronchospasm can assist him in the decision to carry out this additional administration. This would limit the use of this rescue therapy to the bare minimum and, therefore, lower the use of OCS to the lowest effective cumulative dose.

Another potential use is in patients with difficult‐to‐treat asthma, which presents a significant challenge in clinical practice.^46^, ^47^ These patients often require a more intensive study with frequent monitoring and tailored treatment strategies. The app can play a crucial role in determining whether a patient is dealing with severe asthma.

The app can also help identify patterns and triggers associated with severe asthma by providing continuous and precise measurements of bronchial obstruction. This data is invaluable for clinicians in adjusting treatment plans and may aid in distinguishing between true severe asthma and other forms of difficult‐to‐treat asthma, such as those complicated by comorbid conditions.

The GINA recommendations suggest a step‐system for asthma pharmacological management and a crucial moment is the step down.^46^, ^48^ The app may help the physician understand if this intervention was carried out at the correct time and in the correct manner and if the patient’s disease is still controlled even with a lower dosage.

The development of an app capable of detecting bronchial obstruction has significant potential for diagnosing and managing asthma exacerbations. One critical challenge in managing asthma exacerbations is differentiating between bronchospasm and panic attacks, which can present with similar symptoms such as shortness of breath and chest tightness.^49^ Moreover, it is not uncommon for the two diseases to be present at the same time, as it has been shown that anxiety‐related manifestations are more common in asthmatic patients. The app’s ability to detect bronchial obstruction provides an objective measure that can help patients and clinicians distinguish between these conditions.

Allergic but non‐asthmatic patients might also benefit from this tool. For example, a respiratory‐allergic reaction to food often leads to bronchospasm^50^: the app may help distinguish between this medical emergency and subjective fear‐induced throat discomfort.

Observing the potential role of assisting in the diagnostic phase of respiratory obstructive disease management, this tool might be of help in the pediatric population. Children pose unique challenges in respiratory diagnostics, especially when it comes to bronchial provocation tests. Existing methods, such as spirometry and bronchial provocation tests, require a level of cooperation and understanding that can be difficult to achieve with younger patients.^51^ The introduction of an app capable of detecting bronchial obstruction can significantly help mitigate these challenges.

In summary, the app can help patients interpret their symptoms, leading to better management and reduced anxiety. It can also assist clinicians in making more accurate diagnoses, potentially reducing unnecessary treatments and hospital visits. The app’s accessibility could democratize healthcare, improve health outcomes, and reduce asthma management disparities. Its simplicity makes it an ideal tool for research, offering valuable real‐world data that can contribute to a better understanding of asthma and the development of personalized treatment plans. Additionally, the app’s capacity for frequent assessments allows for continuous monitoring and early detection of exacerbations, benefiting patients with unstable asthma. A potential limitation of this study, which we believe we have minimized by excluding patients with signs and symptoms of airway infections, is the fact that we cannot exclude that any subclinical airway infection episodes may have an impact on vocal biomarkers. Validation and assessment of the vocal biomarkers, evaluation of social, environmental, and ethical implications, and ensuring security and interoperability with existing systems are necessary steps before this technology is widely available.

## 5 CONCLUSION

The correlation tests carried out demonstrate the goodness and specificity of some vocal biomarkers assessed by a mobile phone application, in following the variability of FEV_1_ of each single subject. Not surprisingly, the task whose sound achieved the best descriptive power was the one most similar to the performance of a traditional spirometric test. Despite the lack of a single, generalizing biomarker, this work demonstrated the potential of acoustic analysis and encouraged the use of vocal biomarkers in combination with machine learning techniques to find robust models that could connect sound with spirometric parameters. As reported, top correlation values lived in a particularly high range of correlation, regardless of the result of the bronchial challenge test, indeed high descriptive power was found both in asthmatic and non‐asthmatic populations. This suggests that variations in the acoustic dynamics could be related to variations at the level of lower airways. But for practical use and due to statistical instability, subject‐specific features can't be used in a broader application, hence a deeper understanding of acoustic modeling is needed and a tool able to predict pulmonary function with sufficient confidence for every subject must be developed. For this reason, further studies involving additional persons gathered in a bigger dataset must be performed and could enable the realization and evaluation of a general acoustic‐respiratory model.

## AUTHOR CONTRIBUTIONS

**Giovanni Paoletti**: Conceptualization; investigation; writing—original draft; methodology; writing—review and editing. **Giovanni Costanzo**: Writing—original draft; writing—review and editing; investigation. **Morena Merigo**: Investigation; writing—original draft; writing—review and editing. **Francesca Puggioni**: Investigation; writing—original draft; writing—review and editing; conceptualization; methodology. **Sebastian Ferri**: Investigation; writing—original draft; writing—review and editing. **Maria Rita Messina**: Investigation; writing—original draft; writing—review and editing. **Fulvio Cordella**: Conceptualization; funding acquisition; methodology; validation; writing—review and editing; formal analysis; software; data curation. **Giuseppe Ranieri**: Conceptualization; funding acquisition; methodology; validation. **Arianna Arienzo**: Conceptualization; funding acquisition; methodology; validation; software; data curation; project administration. **Victor Savevski**: Writing—original draft; writing—review and editing; investigation. **Giorgio Walter Canonica**: Investigation; writing—original draft; writing—review and editing. **Ayana de Brito Martins**: Conceptualization; funding acquisition; methodology; software; project administration; data curation. **Enrico Heffler**: Supervision; conceptualization; investigation; writing—original draft; writing—review and editing; methodology; validation.

## CONFLICT OF INTEREST STATEMENT

Arianna Arienzo, Ayana de Brito Martins, Giuseppe Ranieri and Fulvio Cordella are shareholders of VoiceMed s.à.r.l. Giovanni Paoletti reports fees for speaker activities and/or advisory board participation from Lofarma, GSK, and AstraZeneca, outside the submitted work. Francesca Puggioni, reports fees for speaker activities and/or advisory boards participation from AstraZeneca, Sanofi, Regeneron, GSK, Menarini, Chiesi, Mundipharma, Valeas, Alk Abelló, Allergy Therapeutics, Boehringer Ingelheim, and Stallergenes Greer outside the submitted work. Giorgio Walter Canonica reports research or clinical trials grants paid to his Institution from Menarini, AstraZeneca,GSK, Sanofi Genzyme and fees for lectures or advisory board participation from Menarini, AstraZeneca, CellTrion, Chiesi, Faes Farma, Firma, Genentech, Guidotti‐Malesci, GSK, HAL Allergy, Innovacaremd, Novartis, OM‐Pharma, Red Maple, Sanofi‐Aventis, Sanofi‐Genzyme, Stallergenes‐Greer and Uriach Pharma, outside the submitted work. Enrico Heffler fees for speaker activities and/or advisory boards participation from Sanofi, Regeneron, GSK, Novartis, AstraZeneca, Stallergenes‐Greer, Chiesi, Almirall, Bosch, Lofarma, outside the submitted work. Giovanni Costanzo, Morena Merigo, Sebastian Ferri, Maria Rita Messina, Victor Saveski reports no conflicts of interest.

## Data Availability

The data that support the findings of this study are available from the corresponding author upon reasonable request.
